# Employment Protection Legislation, Labour Market Dualism, and Fertility in Europe

**DOI:** 10.1007/s10680-023-09662-7

**Published:** 2023-05-04

**Authors:** Elena Bastianelli, Raffaele Guetto, Daniele Vignoli

**Affiliations:** Department of Statistics, Computer Science, Applications, University of Florence, Viale Giovanni Battista Morgagni, 59, 50134 Florence, Italy

**Keywords:** Labour market deregulation, Employment protection legislation (EPL), Fertility, Europe, Fixed-effect estimator

## Abstract

Theoretically, whether a more loosely regulated labour market inhibits or fosters fertility in a society is ambiguous. Empirically, the few studies analysing the relationship between the strictness of employment protection legislation—the norms and procedures regulating labour markets’ hiring and firing processes—and fertility have found mixed evidence. This paper reconciles the ambivalent conclusions of previous studies by analysing the impact of employment protection legislation and labour market dualism on total fertility across 19 European countries between 1990 and 2019. Our results indicate that an increase in employment protection for regular workers positively affects total fertility. Nonetheless, an increasing gap between the regulation of regular and temporary employment—that is, labour market dualism—negatively impacts total fertility. These effects, of small-to-moderate intensity, are relatively homogeneous across age groups and geographical areas and are especially pronounced among the lower educated. We conclude that labour market dualism, rather than a “rigid” employment protection legislation, discourages fertility.

## Introduction

Since the beginning of the 1990s, the relatively strict employment protection legislation (EPL)—the norms and procedures regulating labour markets’ hiring and firing processes (OECD, 1999)—of European labour markets came to be considered one of the main determinants of persistently high unemployment in Europe (Grubb & Wells, 1993). Following the recommendations set by the Organisation for Economic Co-operation and Development (OECD) Jobs Study (OECD, 1994) and the European Employment Strategy to preserve Europe’s competitive edge in a globalized world (Boeri et al., 2012), most European countries undertook a set of ‘deregulatory’ reforms to increase labour market flexibility (Cutuli & Guetto, 2013). Reforms mainly included the progressive liberalization and promotion of new forms of flexible and temporary work contracts, characterized by lower bargaining power, lower levels of social protection, and generally lower wages (Barbieri & Cutuli, 2018). While the diffusion of flexible working contracts has contributed to reducing unemployment, at least in the short run, it has also caused increasing precariousness of career paths, with negative consequences on individuals’ life courses (Garibaldi & Taddei, 2013; Kim et al., 2008; Scherer, 2009). By affecting labour market entry and exit conditions and individuals’ job security, deregulation reforms may affect family formation (Cerruti et al., 2022; de Paola et al., 2021; Karabchuk, 2020).

The literature on the effect of an individual’s labour market situation on family formation has often focused on single episodes of unemployment or temporary employment over one’s life course, by means of event history analyses modelling the effects of labour market transitions, for example, job losses or shifts from fixed-term to permanent contracts. A consistent stream of studies has shown that flexible and temporary work contracts delay the transition to adulthood and lead to fertility postponement and decline (Barbieri et al., 2015; Dantis & Rizzi, 2020; Kreyenfeld et al., 2012; Pailhé & Solaz, 2012; Vignoli et al., 2012, 2016, 2020a, 2020b, 2020c). However, the time-to-event empirical strategy adopted in these studies makes it difficult to evaluate the overall fertility consequences of the several waves of labour market deregulation reforms implemented in Europe. In addition, most existing studies focus on the transition to the first child, whereas higher-order childbirths are rarely analysed. This study addresses these oversights in previous research by focusing on the impact of labour market (de)regulation reforms on total fertility across Europe.

Whether a more flexible EPL inhibits or fosters fertility at the aggregate level is theoretically ambiguous, and the empirical evidence is mixed. On the one hand, labour market deregulation has been shown to decrease job tenure, increase unemployment inflows, and increase perceptions of job insecurity (Anderson & Pontusson, 2007; Cazes & Tonin, 2010). Thus, it may render it more difficult for individuals to make long-term plans and lead potential parents to postpone fertility (de Paola et al., 2021; Fahlén & Oláh, 2018; Prifti & Vuri, 2013; ). On the other hand, however, more liberal labour legislation may facilitate the recruitment of young workers, leading to lower youth unemployment rates (Bertola et al., 2007; Hipp et al., 2015), and consequently higher fertility (Adsera, 2011; Karabchuk, 2020). Only a handful of studies have considered the effects of EPL on fertility in Europe and have found evidence for both mechanisms (e.g. Adsera, 2005; Bellani, 2020; Cerruti et al., 2022; de Paola et al., 2021; Rovny, 2011). Thus, the evidence is mixed, and policy implications are unclear.

In this article, we advance that these ambiguous theoretical expectations and contrasting evidence regarding the impact of EPL on fertility can be reconciled by considering that labour market reforms have been mostly “partial and targeted” (Esping-Andersen & Regini, 2000). In many European countries, deregulatory reforms only applied to new jobs and mainly affected a disadvantaged fraction of the population (primarily youths and low-skilled workers), while legislation for regular contracts remained substantially unchanged for a long time (Barbieri & Scherer, 2009; Boeri & Garibaldi, 2007; Boeri et al., 2012). The reforms did not substantially alter the overall employment rates while generating increasing labour market dualism (Barbieri & Cutuli, 2016; Gebel & Giesecke, 2016). In a dual labour market, it is more difficult and time-demanding for young people to fully integrate into the market (Barbieri & Cutuli, 2016), with plausible negative consequences for their reproductive behaviours. Our main argument is that the gap between the regulation of regular and temporary employment—that is, labour market dualism—rather than a rigid EPL depresses fertility. By contrast, increasing employment protection and reducing employment uncertainty should foster higher fertility rates, in line with the micro-level evidence suggesting that stable employment is a prerequisite for fertility.

Building on this argument, we aim to evaluate the impact of protecting regular contracts and of the changing differentials in EPL between regular^1^ and temporary workers, the so-called EPL-gap (Barbieri & Cutuli, 2016; Bentolila et al., 2012), on total fertility. We ask whether deregulation reforms and, specifically, marginal EPL reforms that increased the gap in protection levels between distinct segments of the labour force led to negative fertility consequences. Moreover, we ask whether EPL has a different impact on the fertility of specific population subgroups, defined in terms of age and education, or across countries.

## Labour Market (De)Regulation and Fertility

EPL includes all norms regulating labour markets’ hiring and dismissing processes; for example, EPL includes conditions for using temporary contracts, redundancy procedures, mandated prenotification periods and severance payments, special requirements for collective dismissals, and so forth. It refers to all types of employment protection measures, whether grounded primarily in legislation, court rulings, collectively bargained conditions of employment, or customary practice (OECD, 1999). The degree of labour market regulation is generally measured separately for regular and temporary work and measures the strictness of labour laws on dismissing permanent employees and opportunities for employers to hire workers on a temporary basis (OECD, 2013). However, when measuring labour market flexibility/rigidity, the literature generally refers to the degree of employment protection for regular workers. A *rigid* (*strict*) EPL guarantees strong employment protection for regular workers, while a *flexible* (or *liberal*, *loose*) EPL allows more unstable jobs.

By influencing individuals’ job stability, EPL has the potential to influence fertility (de Paola et al., 2021; Karabchuk, 2020). However, it is theoretically ambiguous whether a flexible EPL inhibits or fosters fertility in a society. By increasing labour market mobility, a flexible EPL may generate employment uncertainty, making it more difficult for individuals to predict their future and make long-term plans, thus leading them to postpone fertility (de Paola et al., 2021; Fahlén & Oláh, 2018; Prifti & Vuri, 2013). The primary purpose of EPL is indeed to provide workers with more stability in their current jobs. A strict EPL has been found to increase job tenure (Cazes & Tonin, 2010) and reduce the inflow rate into unemployment (OECD, 2004). Furthermore, a rigid EPL increases the perceived stability and continuity of one’s employment. With stricter EPL, individuals have been found to be less worried about losing their jobs and to perceive their jobs as more secure, whereas a weakening in EPL was associated with higher employment uncertainty (Anderson & Pontusson, 2007). Uncertainty over employment may lead young people to defer family formation until full integration into the labour market is achieved (Bolano & Vignoli, 2021) and, ultimately, have fewer children than desired (van Wijk et al., 2021). As a matter of fact, in the field of fertility research, various micro-level studies have shown that employment instability (i.e. unemployment and/or temporary working contracts) contributes to childbearing postponement and reduction (Alderotti, 2022; Alesina et al., 2015; Barbieri et al., 2015; Busetta et al., 2019; Kreyenfeld & Andersson, 2014; Özcan et al., 2010; Pailhé & Solaz, 2012; Vignoli et al., 2012, 2020a, 2020b, 2020c; for a meta-analysis of European research findings see: Alderotti et al., 2021). Thus, *a deregulated labour market may hinder fertility rates*.

However, the literature also reports that a rigid EPL hampers young people’s integration into the labour market (Hipp et al., 2015). Extensive protection for permanent workers increases the costs of hiring young labour market entrants with no experience and incentivizes firms to offer them temporary jobs (Cazes & Tonin, 2010; Kahn, 2007). A rigid EPL reduces labour market dynamics; that is, it decreases both the inflow rate into unemployment and the rate of exit from unemployment, with detrimental consequences for the (re-)employment chances of the young and especially of young women with intermittent labour market participation (Bertola et al., 2007; OECD, 2004). With rigid EPL, it is generally difficult and costly to fire workers, with the effect that, even in good times, firms tend to hire fewer workers because these workers may become redundant in the future (Montenegro & Pagés, 2004). Wrongful-discharge protection laws, for instance, raising employment costs without yielding corresponding productivity increases, have been found to reduce employment rates, especially for women and less educated workers who change jobs more frequently (Autor et al., 2006). By contrast, more liberal labour legislation corresponds to higher flexibility in recruitment and dismissals. It follows that a flexible EPL is usually associated with lower barriers to entering the labour market and lower youth unemployment rates (Bertola et al., 2007; Hipp et al., 2015). As youth unemployment hampers family planning (Comolli, 2017), *a deregulated labour market may foster fertility rates*.

The empirical evidence on the relationship between EPL and fertility is limited and supports both perspectives. Fahlén and Oláh (2018) found that short-term fertility intentions have increased in countries where EPL has been strengthened from 2004 to 2011. Furthermore, studies analysing the causal impact of two different EPL reforms in Italy found that the reduced employment instability following a strengthening of EPL had a positive and sizable effect on the fertility behaviours of Italian working women (Prifti & Vuri, 2013), while a subsequent reduction in job stability significantly lowered women’s propensity to have a child (de Paola et al., 2021). In addition, higher employment protection has been shown to make it easier for women to combine work and family responsibilities (Bratti et al., 2005) and to increase life satisfaction (Ochsen & Welsch, 2012), with plausible positive effects on fertility (Mencarini et al., 2018; Vignoli et al., 2020a, 2020b, 2020c). Nevertheless, many studies analysing the relationship between EPL and fertility found a rigid EPL to be associated with lower fertility rates (Adsera, 2004, 2005, 2011; Bellani, 2020; Rovny, 2011) or not to have any significant effect (Karabchuk, 2020; Luci-Greulich & Thévenon, 2013; Vignoli et al., 2020a, 2020b, 2020c). Importantly, most of these studies only consider EPL as a confounding factor within models designed to test other research hypotheses.

We argue that the ambiguous theoretical expectations and empirical results concerning the association between EPL and fertility can be addressed by disentangling EPL into its two components related to regular and temporary employment. In fact, the (few) existing studies on the link between EPL and fertility ignore that the EPL reforms of the last 3 decades mostly affected employment patterns by creating labour market dualism through marginal and/or targeted provisions that only apply to new jobs. Such reforms mostly penalize labour market entrants, and especially the youth, women and low-skilled workers with intermittent employment spells (Barbieri & Cutuli, 2016; Cazes & Tonin, 2010). A bulk of research has shown that the degree of rigidity/flexibility of EPL does not substantially alter the employment and unemployment rates in a country; rather, deregulating temporary contracts while leaving the protection of regular contracts unchanged—that is, increasing the EPL-gap—is responsible for rising youth unemployment rates and the diffusion of more precarious forms of employment (Barbieri & Cutuli, 2016; Bentolila et al., 2012; Boeri & Garibaldi, 2007; Gebel & Giesecke, 2016; Noelke, 2016). With a wide EPL-gap, the proportion of temporary jobs converted into permanent jobs diminishes. Indeed, high firing costs for permanent workers coupled with loose restrictions on the use of temporary contracts induce employers to use temporary jobs in sequence rather than converting them into permanent ones (Bentolila et al., 2012). Thus, facilitating the widespread use of flexible temporary contracts in labour markets already regulated by stringent permanent job security provisions only has a transitional “honeymoon” job-creating effect (Boeri & Garibaldi, 2007), while generating low job mobility between the “outsider” and “insider” segments of the labour market (Barbieri & Cutuli, 2018). In dual labour markets, the perceived job insecurity of the “outsider” contingent is significantly higher (Balz, 2017), and it is harder and takes longer for young people to fully integrate into the world of work, with negative consequences for their well-being (Voßemer et al., 2018) and, presumably, their reproductive choices.

We thus posit that the EPL-gap, that is, the differential in employment protection between regular and temporary workers, rather than a rigid EPL per se, discourages fertility. Specifically, we hypothesize that:

### **H1**

Increasing labour market protections for regular contracts (EPL-r) fosters higher fertility rates.

By contrast,

### **H2**

Increasing labour market dualism (EPL-gap) leads to lower fertility rates.

## Employment Protection Legislation and Fertility: Heterogeneity Across Age, Educational Groups, and Country Contexts

Group-specific differences—for example, age and education—in the impact of EPL on fertility have seldom been considered in previous research. Nevertheless, research on the impact of EPL on employment patterns has found that the degree of rigidity/flexibility of EPL has different effects across subgroups of workers (Autor et al., 2006; Gebel & Giesecke, 2016; Kahn, 2007; Montenegro & Pagés, 2004). For instance, it has been found that job security provisions reduce the employment rates of youth and the unskilled at the benefit of older and skilled workers, and tend to benefit men at the expense of women (Montenegro & Pagés, 2004). Moreover, as in dual labour markets the young, women, and the unskilled are more likely to be “outsiders”, then these groups are those more likely to bear the negative consequences of increases in the EPL-gap. Thus, EPL may differentially impact the fertility of different population subgroups.

Regarding age, strict protection for regular contracts hampers labour market entrance for young workers with no experience (Cazes & Tonin, 2010; Hipp et al., 2015). It follows that labour market entrants could benefit relatively less from a strict EPL also in terms of fertility. Young adults were also the most affected by deregulation reforms, as deregulation mainly applied to new jobs (Gebel & Giesecke, 2016; Noelke, 2016). Thus, labour market entrants may postpone their fertility until they reach more stable employment conditions. The negative effects of the EPL-gap on fertility may be felt only at relatively young ages, and a fertility catch-up could occur at older ages once employment stability is achieved. Nevertheless, fully integrating into a dual labour market may be time-consuming, and there may not be time for catching up on fertility. In addition, it is not straightforward which age group defines the “labour market entrants”, as it may vastly vary according to individuals’ level of education and country context (e.g. educational system or culture).

Thus, in this research, *we test whether increasing strictness in EPL for regular workers and increasing labour market dualism have different effects on the fertility of different age groups*, without advancing specific hypotheses.

Concerning education, in dual labour markets, low-skilled workers face a higher risk of becoming unemployed (Gebel and Giesecke, 2016), while the highly educated have been found to benefit more from stricter EPL in terms of occupational status attainment (Wolbers, 2007). Therefore, the highly educated could benefit more from a rigid EPL also in terms of fertility outcomes. By contrast, as labour market dualism mainly affects low-skilled workers, the lower educated may face worse negative consequences of an increasing EPL-gap on fertility. Thus, we expect that increasing strictness in the EPL for regular workers and the widening of the EPL-gap will have different impacts on the fertility of different educational groups.

In particular, we expect that *the positive effect of an increase in EPL-r on fertility will be stronger for highly educated women* (Hypothesis 1a), while *the negative effect of an increase in the EPL-gap will be stronger for the lower educated* (Hypothesis 2a).

The effects of labour market reforms may also vary across institutional contexts (Balz, 2017). For instance, labour market policies (such as unemployment benefits or assistance in job search) may influence the relationship between employment instability and fertility by affecting unemployment duration or opportunities for entering stable employment, or by providing financial support in the case of unemployment (Adsera, 2004, 2005; OECD, 2006). Nordic countries are known for providing strong welfare support and implementing active labour market policies that facilitate (re-)entry into employment (Esping-Andersen, 1999). Continental countries also provide strong financial support for the unemployed. In the Anglo-Saxon area, labour markets are highly flexible with relatively short unemployment spells (Adsera, 2004). Social assistance for the unemployed is the least generous in Southern Europe and post-socialist countries of Central and Eastern Europe (Esping-Andersen, 1999; Javornik, 2014). In addition, Southern countries are characterized by high levels of youth unemployment, temporary employment, and involuntary part-time employment (Barbieri & Scherer, 2009; Barbieri et al., 2019).

Considering this substantial variation in welfare regimes and labour market contexts in Europe, it is difficult to advance specific hypotheses; however, in this study, *we account for possible heterogeneity in the impact of EPL on fertility across different country-areas.*

## The Different Waves of Labour Market (De)Regulation in Europe

In Europe, most employment protection norms in the modern form were developed through legislation, collective agreements, and court rulings between the 1960s and 1980s. The process of increasing employment protection through the regulation of hiring and firing dynamics reached relatively regulatory stability during the 1980s (OECD, 2013). Since the beginning of the 1990s, most European countries undertook a set of ‘deregulatory’ reforms to increase labour market flexibility. This first wave of labour market deregulation mainly concerned the drastic deregulation of hiring on temporary contracts while maintaining restrictions on regular contracts (Cutuli & Guetto, 2013; Garibaldi & Taddei, 2013). Other than Anglo-Saxon countries that have always allowed the use of temporary contracts without any specific reason, in many countries, there was a substantial relaxation of the regulations regarding the use of temporary contracts (OECD, 1999). The most prevalent path of reform involved facilitating the use of fixed-term contracts and recourse to workers hired from temporary work agencies (OECD, 2004). For instance, reforms at the beginning of the 1990s in Belgium, Germany, and Sweden made fixed-term contracts possible without specifying objective reasons. Moreover, in Germany and Belgium, the number of possible renewals and overall duration of temporary contracts were progressively widened. Between the end of the 1990s and the early 2000s, temporary work agencies were liberalized in Italy and Spain, as well as different types of temporary work contracts (OECD, 1999, 2004).

A tendency towards the deregulation of regular contracts began at the end of the first decade of the 2000s, following the onset of the financial crisis (OECD, 2013). The action was mostly taken in countries where legislation for regular contracts was stricter, as in southern Europe, but also in other areas. For instance, between 2009 and 2014, various reforms shortened notice periods in Portugal, Slovakia, Greece, and Spain. Furthermore, the required severance pay for dismissals was reduced in Portugal and Greece. In Portugal, the range of valid grounds for termination was increased, making dismissals of regular contracts easier. In Italy, the eligible cases in which reinstatement could be ordered by the court were restricted to only discrimination. Moreover, in the United Kingdom, where regulation was already relatively loose, the minimum period between notification to the administration and a collective dismissal was halved (OECD, 2013).

Another wave of deregulation reforms occurred in the most recent period (2014–2019) in an attempt to reduce labour market dualism (OECD, 2019). Recent reforms can be classified into two main categories. The first class of reforms continued in the direction of reducing the restrictions on dismissing regular workers (sometimes at the same time as they eased restrictions on the use of temporary employment). Among those, major reforms involving several aspects of regulation took place in France, Italy, and Slovenia. The second group of reforms instead increased restrictions on the use of temporary employment. For instance, several countries (e.g. Poland, Germany, and Slovakia) introduced a legal limit for the cumulative duration of fixed-term contracts or temporary work agency assignments. Others (e.g. Italy and Denmark) introduced the obligation to provide a rationale when using a fixed-term contract in certain circumstances (OECD, 2019).

## Data and Trends

To analyse the impact of EPL on fertility, we built a country-level panel with 19 European countries (Austria, Belgium, Czech Republic, Denmark, Finland, France, Germany, Greece, Hungary, Ireland, Italy, Netherlands, Norway, Poland, Portugal, Slovakia, Spain, Sweden, and UK)^2^ covering the period between 1990 and 2019.^3^

Our dependent variable is the countries’ total fertility rate (TFR) provided by OECD. To test for heterogeneity across age and educational groups, we additionally used age- and education-specific fertility rates as dependent variables. Age-specific fertility rates are provided by Eurostat and are available for all countries and years except for France and Germany, where they are available beginning in 1998 and 2000, respectively. Education-specific fertility rates have been calculated from Eurostat data. The rates are based on the number of live births by the mother’s level of education (15–39 years old) divided by the total number of women by the level of education (15–39 years old) for each country and year. Information is only available from 2007 to 2019,^4^ and only for 13 countries. The information is missing for France, Germany, Ireland, Italy, the Netherlands, and the UK.

The main explanatory variables are derived from the OECD employment protection indexes for regular and temporary workers. The OECD indexes are compiled from 21 items covering different aspects of employment protection regulations in a country, as they were in force on 1 January of each year. Information is collected from detailed questionnaires completed by local experts, government authorities, and the OECD Secretariat, integrated with national and international secondary sources (e.g. statutory laws, collective bargaining agreements, and case law). Then, it is converted into a score measured on a 0–6 scale, with higher values representing stricter regulation (OECD, 1999, 2013). The EPL index for regular workers measures the strictness of the labour laws on firing permanent employees in a country. It incorporates three main aspects of dismissal protection: (i) procedural inconveniences that employers face when initiating the dismissal process (e.g. notification and consultation requirements), (ii) notice periods and severance pay, and (iii) circumstances in which it is possible to dismiss workers and the repercussion on employers for unfair dismissals (OECD, 2013). The EPL index for temporary contracts concerns, instead, the possibility and conditions for employers to hire workers on a temporary basis, including the types of work for which these contracts are allowed and the possibilities for their renewal and cumulative duration (OECD, 1999, 2013). In our models, the main independent variables are the EPL index for regular workers (EPL-r) and the EPL-gap, that is, the difference between the two indexes.^5^ The variable measuring the EPL-gap ranges from − 3 to 4 in our analytical sample. Changes in EPL-r scores correspond to reforms in EPL regarding regular contracts, while changes in EPL-gap scores correspond to changes in the gap between the regulation of regular and temporary contracts.

The OECD EPL indexes are the best available comparative indicators of employment protection legislation. The OECD puts extensive efforts into making these indicators more and more valid and reliable (OECD, 2004, 2013, 2019), and are widely employed in scientific research and policy evaluation. However, they do have a series of limitations (Bertola et al., 2000; Boeri & Jimeno, 2005; Myant, 2016). First, they rely heavily on subjective assessments, which can make cross-country comparability problematic. Moreover, they rely on formal legislation, which may not be enforced or may be enforced unevenly. Finally, they are limited to formal employment, making it problematic as a measure of de facto labour market regulation in countries with a large informal sector (Myant, 2016). Nevertheless, these issues are a matter of concern mainly when considering cross-country differences in the levels of regulation, while they may be less problematic in a within-country, over-time approach.

**Fig. 1:**
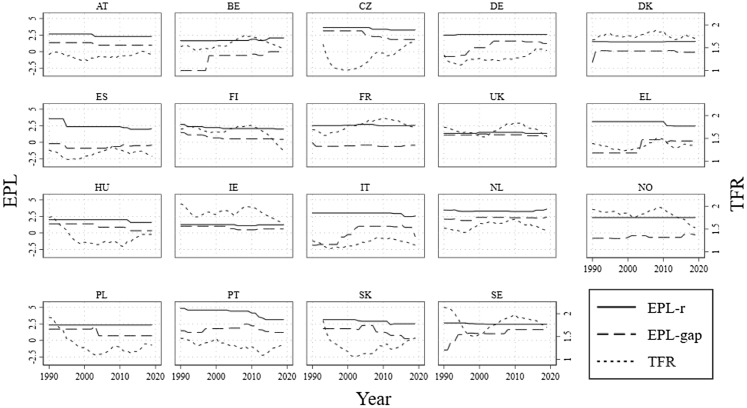
EPL-r, EPL-gap, and TFR for each country

Figure 1 displays the countries’ EPL-r, EPL-gap, and fertility trends. In 2019, the EU TFR stood at 1.53. Among the countries included in this study, the one with the highest TFR in 2019 was France (1.86), followed by the Czech Republic, Ireland, and Sweden (1.71), then Denmark (1.70). The lowest fertility rates were observed in Spain (1.23), Italy (1.27), and Greece (1.34). Over the entire period, the EPL-gap appears to increase in most countries, while the EPL for regular contracts remained substantially unchanged, with some declines since the 2010s. This does not necessarily mean that there were no changes in employment legislation for regular contracts at all. Legislation may have changed, but it did not come with such changes as to suggest the national experts and the OECD secretariat a shift in the overall level of protection. These descriptive results confirm that most countries undertook partial EPL deregulation, whereby the use of temporary contracts was liberalized while the protection of regular contracts was minimally changed. For some countries, a reduction in the gap is evident in the last decade due to the previously mentioned reforms aimed at reducing labour market dualism.

## Methodology

We empirically estimated the impact of EPL on fertility with a fixed-effect (FE) estimator, that is, by analysing within-country variations. Formally, our model can be described as follows:

1 $$\widetilde{{{\text{TFR}}_{{{\text{ct}}}} }} = \beta_{1} \widetilde{{{\text{EPLr}}_{{{\text{ct}}}} }} + \beta_{2} \widetilde{{{\text{EPLgap}}}}_{{{\text{ct}} }} + T_{{\text{t}}} + C_{{\text{c}}} *T_{{\text{t}}} + T_{{\text{t}}}^{2} + C_{{\text{c}}} *T_{{\text{t}}}^{2} + \widetilde{{\varepsilon_{{{\text{ct}}}} }}$$

All variables are country de-meaned (as indicated by the tildes) to capture within-country variation. The element $T_{{\text{t}}} + C_{{\text{c}}} *T_{{\text{t}}} + T_{{\text{t}}}^{2} + C_{{\text{c}}} *T_{{\text{t}}}^{2}$ represents country-specific linear and quadratic time trends, and $\widetilde{\varepsilon_{{{\text{ct}}}}}$ represents country-specific error terms (*c* and *t* in the subscripts stand for country and time). $\widetilde{{{\text{EPLr}}_{{{\text{ct}}}} }}$ and $\widetilde{{{\text{EPLgap}}}}_{{\text{ct }}}$ refer to the legislation in force on 1 January and are 1-year lagged relatively to the TFR.

One of the problems with the current literature on EPL effects is that most of the evidence is based on cross-country time-series data, which is generally affected by endogeneity and measurement problems (Barbieri & Cutuli, 2016; Bertola, 2014; Hijzen et al., 2013). The FE estimator, performing regressions on the deviations from the country means, disentangles the impact of changes in the EPL-r and EPL-gap from that of time-constant country characteristics that affect fertility. By using a FE model, we addressed potential bias due to unobserved time-constant country characteristics, such as national culture and the institutional and economic environment. We compared the results of the FE model with those of the between-effect and random-effect models and found the FE model to be superior based on the results of the Hausman test. Furthermore, the model includes country-specific linear and quadratic time trends to capture the underlying fertility trend in each country, thus accounting for country-level time-varying unobserved heterogeneity. This analytic strategy reduces the risk of omitted variable bias and takes into account that fertility may have evolved in different ways across countries for reasons other than EPL reforms. Including country-specific time trends is thus necessary to uncover the impact of EPL-r and EPL-gap on fertility. We added the quadratic term as fertility trends did not evolve linearly in the period observed.

Then, we augmented Eq. (1) with a set of control variables that have been found to influence fertility and may confound the effects of EPL-r and EPL-gap: a first group related to family policies, i.e. the number of weeks of paid maternity leave, and public spending on the family as a percentage of GDP^6^; and a second group, i.e. unemployment and women’s employment rates, accounting for labour market characteristics (Adsera, 2004; Comolli, 2017; Matysiak et al., 2021). All independent variables are 1-year-lagged relatively to fertility. Controlling for these time-varying variables, we can distinguish the effects of EPL-r and EPL-gap on fertility from those of other competing macro-level factors. We thus obtained the following equation:

2 $$\widetilde{{{\text{TFR}}_{{{\text{ct}}}} }} = \beta_{1} \widetilde{{{\text{EPLr}}_{{{\text{ct}}}} }} + \beta_{2} \widetilde{{{\text{EPLgap}}}}_{{\text{ct }}} + \beta_{3} \widetilde{{{\text{MatLeave}}}}_{{{\text{ct}}}} + \beta_{4} \widetilde{{{\text{PubSpe}}}}_{{{\text{ct}}}} + \beta_{5} \widetilde{{{\text{Unempl}}}}_{{{\text{ct}}}} + \beta_{6} \widetilde{{{\text{WEmpl}}}}_{{{\text{ct}}}} + T_{{\text{t}}} + C_{{\text{c}}} *T_{{\text{t}}} + T_{{\text{t}}}^{2} + C_{{\text{c}}} *T_{{\text{t}}}^{2} + \widetilde{{\varepsilon_{{{\text{ct}}}} }}$$

The unemployment rate and women’s employment rate are also influenced by EPL and, thus, may mediate its effect. Therefore, as a robustness check (Sect. 7.2), we ran additional models introducing different temporal lags to account for the ordering of effects.

To test for heterogeneity across age and educational groups (Sect. 7.1), we estimated models with age-specific and education-specific fertility rates as dependent variables, respectively, while the right-hand side of equations as in Eq. (1).

Our FE models analyse within-country variation assuming a similar effect of EPL-r and EPL-gap across countries. However, the effects of labour market reforms may vary across institutional contexts (Balz, 2017). To investigate possible cross-country heterogeneity, we tested for interaction effects between EPL-r and EPL-gap and European areas (i.e. Anglo-Saxon, Continental, Southern, Central and Eastern, and Nordic).^7^ In this supplementary analysis, we included country-area dummies and their interactions with EPL-r and EPL-gap. Thus, in order to avoid overspecification and keep substantial variability in the model, variables were not country de-meaned, and country-specific time trends were replaced with year fixed effects.^8^

3 $${\text{TFR}}_{{{\text{ct}}}} = \beta_{1} {\text{EPLr}}_{{{\text{ct}}}} + \beta_{2} {\text{EPLgap}}_{{\text{ct }}} + {\text{EPLr}}_{{{\text{ct}}}} *A_{{\text{a}}} + {\text{EPLgap}}_{{{\text{ct}}}} {*}A_{{\text{a}}} + A_{{\text{a}}} + \beta_{3} {\text{MatLeave}}_{{{\text{ct}}}} + \beta_{4} {\text{PubSpe}}_{{{\text{ct}}}} + \beta_{5} {\text{Unempl}}_{{{\text{ct}}}} + \beta_{6} {\text{WEmpl}}_{{{\text{ct}}}} + T_{{\text{t}}} + \varepsilon_{{{\text{ct}}}}$$

In Eq. (3), $\beta_{1} {\text{EPLr}}_{{{\text{ct}}}}$ and $\beta_{2} {\text{EPLgap}}_{{\text{ct }}}$ are the main effects of EPL-r and EPL-gap, ${\text{EPLr}}_{{{\text{ct}}}} *A_{{\text{a}}}$ is the interaction term between EPL for regular contracts and country-areas, ${\text{EPLgap}}_{{{\text{ct}}}} *A_{{\text{a}}}$ is the interaction term between EPL-gap and country-areas, $A_{{\text{a}}}$ denotes country-area fixed effects, and $T_{{\text{t}}}$ represents year fixed effects (the subscripts *a* and *t* stand for country-area and time).

Several robustness checks are provided in Sect. 7.2. Descriptive statistics and data sources for all variables included in the analysis can be found in Table 4 in the Appendix.

## Results

Model 1 in Table 1 provides the linear effect of a unit increase in EPL-r and EPL-gap on the within-country TFR change over time, accounting for time-invariant country characteristics and country-specific time trends, as in Eq. (1). The coefficients of the two EPL variables support hypotheses 1 and 2: following an increase in employment protection for regular workers (EPL-r), we see an increase in the TFR, while an increase in labour market dualism (EPL-gap) leads to lower fertility rates. The estimated coefficients suggest a considerable positive impact of a unitary increase in EPL-r on fertility, which increases fertility rates by 0.14. A one-point increase in the EPL-gap, instead, leads to a reduction in fertility of about 0.04. Nevertheless, considering that EPL-r varies on a 0–6 scale (− 6, 6 the EPL-gap) and withincountry changes are limited, a one-point-change in EPL-r is to be regarded as a considerable change. Thus, its effect on fertility is significant but relatively small.

**Table 1:** Fixed-effect regression on TFR

Variables	(1)	(2)	(3)	(4)
		Random effect	FE + family policy controls	+ labour market controls
EPL-r	0.137***	0.001	0.122***	0.018
	(0.035)	(0.035)	(0.035)	(0.031)
EPL-gap	− 0.037***	− 0.041***	− 0.033***	− 0.041***
	(0.013)	(0.013)	(0.012)	(0.011)
Weeks of paid maternity leave			0.000	0.001**
			(0.000)	(0.000)
Public spending on the family			0.086***	0.164***
			(0.016)	(0.015)
% Unemployment				− 0.015***
				(0.001)
% Women employment				0.012***
				(0.004)
Country-specific time trends	Yes	Yes	Yes	Yes
Observations	545	545	517	501
*R*-squared	0.625	0.849	0.616	0.724
Number of countries	19	19	19	19

Standard errors in parentheses. ***p < 0.01, **p < 0.05, *p < 0.1. Countries: Austria, Belgium, Czech Republic, Denmark, Finland, France, Germany, Greece, Hungary, Ireland, Italy, Netherlands, Norway, Poland, Portugal, Slovakia, Spain, Sweden, UK. All covariates are 1-year lagged relatively to the TFR

For comparison, Model 2 displays the results of the same model obtained with a random-effect (RE) estimator. Even with this specification, that also includes the between-country variation, a wider gap in the protection of regular and temporary workers is associated with lower fertility rates, while the effect of EPL-r is virtually null. Nevertheless, due to the limitations of the EPL indexes, employing between-country variation is likely to produce biased results due to endogeneity and measurement problems. Moreover, the Hausman test confirmed that the hypothesis that the country-level effects are adequately modelled by a random-effect model is resoundingly rejected (*p* value 0.000). Thus, in Model 3, we return to a FE estimation, and we add our controls for family polices, i.e. the number of weeks of paid maternity leave, and public spending on the family. Accounting for these two variables, the effects of EPL-r and EPL-gap remain statistically significant and of a similar magnitude as in Model 1. Finally, in Model 4 we also account for labour market characteristics, i.e. the unemployment rate and women’s employment rate. Adding these two variables to our model, the effect of EPL-r shrinks and loses statistical significance, while the effect of EPL-gap remains stable. Indeed, unemployment and women’s employment may also depend on EPL. The effect of EPL-r, therefore, could be captured by changes in unemployment and women’s employment.

Overall, EPL seems to exert, on average, a small-to-moderate impact on fertility rates in Europe. Moreover, in line with existing research, our estimated coefficients for the control variables confirm that increases in paid maternity leave, public spending on family benefits, and women employment, positively affect fertility rates, while rising unemployment leads to lower fertility rates (Adsera, 2011; Comolli, 2017; Luci-Greulich & Thévenon, 2013; Matysiak et al., 2021; Rovny, 2011).

### Heterogeneity Across Age, Educational Group, and Country Contexts

To inspect possible differences across age groups, we estimated our model, specified as in Eq. (1), on age-specific fertility rates for the following age groups: 15–24, 25–29, 30–34, 35–39, and 40+. Results are displayed in Table 2. Age-specific fertility rates are multiplied by 10 to improve readability. Our results show that increased protection for regular workers positively affects the fertility of all age groups (although the effect is not statistically significant for the 30–34 age group). The effect is stronger in magnitude for younger age groups (15–24 and 25–29).^9^ Thus, we do not find evidence for the idea that very young age groups are penalized, in terms of fertility, by a stricter EPL-r; on the contrary, it appears that they benefit the most from it.

**Table 2:** EPL and age-specific fertility

Variables	15–24	25–29	30–34	35–39	40+
EPL-r	0.062***	0.080***	0.031	0.037***	0.006***
	(0.011)	(0.021)	(0.022)	(0.011)	(0.001)
EPL-gap	− 0.007	− 0.016**	− 0.030***	− 0.015***	− 0.002***
	(0.004)	(0.008)	(0.008)	(0.004)	(0.001)
Country-specific time trends	Yes	Yes	Yes	Yes	Yes
Observations	528	528	528	528	528
*R*-squared	0.951	0.839	0.895	0.959	0.969
Number of countries	19	19	19	19	19

Standard errors in parentheses. ***p < 0.01, **p < 0.05, *p < 0.1. Countries: Austria, Belgium, Czech Republic, Denmark, Finland, France, Germany, Greece, Hungary, Ireland, Italy, Netherlands, Norway, Poland, Portugal, Slovakia, Spain, Sweden, UK. EPL variables are 1-year lagged relatively to the TFR. Age-specific fertility rates are multiplied by 10

Similarly, increasing the gap in employment protection between regular and temporary workers leads to a reduction in fertility for all age groups. Although we do not observe cohort progression, our results suggest that increasing EPL-gap reduces completed fertility rather than just inducing fertility postponement. The reduction in fertility due to EPL-gap is smaller and not statistically significant for the youngest age group (15–24); its largest effect is found in the 30–34 age group.^10^ Thus, the 30–34 age group appears to be the one most affected by marginal labour market deregulation.

Next, we assessed the fertility effects of changes in EPL-r and EPL-gap for lower-, middle- and highly educated women. Results are shown in Table 3. It is important to note that education-specific fertility data are only available for the period 2007–2019 and for only 13 countries, which implies a substantial loss of country-year observations (154 instead of 545). As for age-specific fertility rates, education-specific fertility rates are multiplied by 10 to improve readability. Increased protection for regular contracts has a positive and significant effect on all educational groups. Thus, in contrast with our hypothesis 1a, our results suggest that highly educated individuals do not benefit more than others from stricter employment regulations in terms of fertility. Conversely, in line with our expectations (hypothesis 2a), increasing labour market dualism has a negative and significant impact only on the fertility of lower-educated women. This result is in line with the argument that the lower-educated are more strongly affected by labour market dualism.

**Table 3:** EPL and education-specific fertility

Variables	Low	Mid	High
EPL-r	0.100**	0.071*	0.090**
	(0.040)	(0.036)	(0.041)
EPL-gap	− 0.074***	− 0.017	0.003
	(0.027)	(0.024)	(0.027)
Country-specific time trends	Yes	Yes	Yes
Observations	154	154	154
*R*-squared	0.572	0.732	0.746
Number of countries	13	13	13

Standard errors in parentheses. ***p < 0.01, **p < 0.05, *p < 0.1. Countries: Austria, Belgium, Czech Republic, Denmark, Finland, Greece, Hungary, Norway, Poland, Portugal, Slovakia, Spain, Sweden. Period: 2007–2018. EPL variables are 1-year lagged relatively to the TFR. Education-specific fertility rates are multiplied by 10

**Fig. 2:**
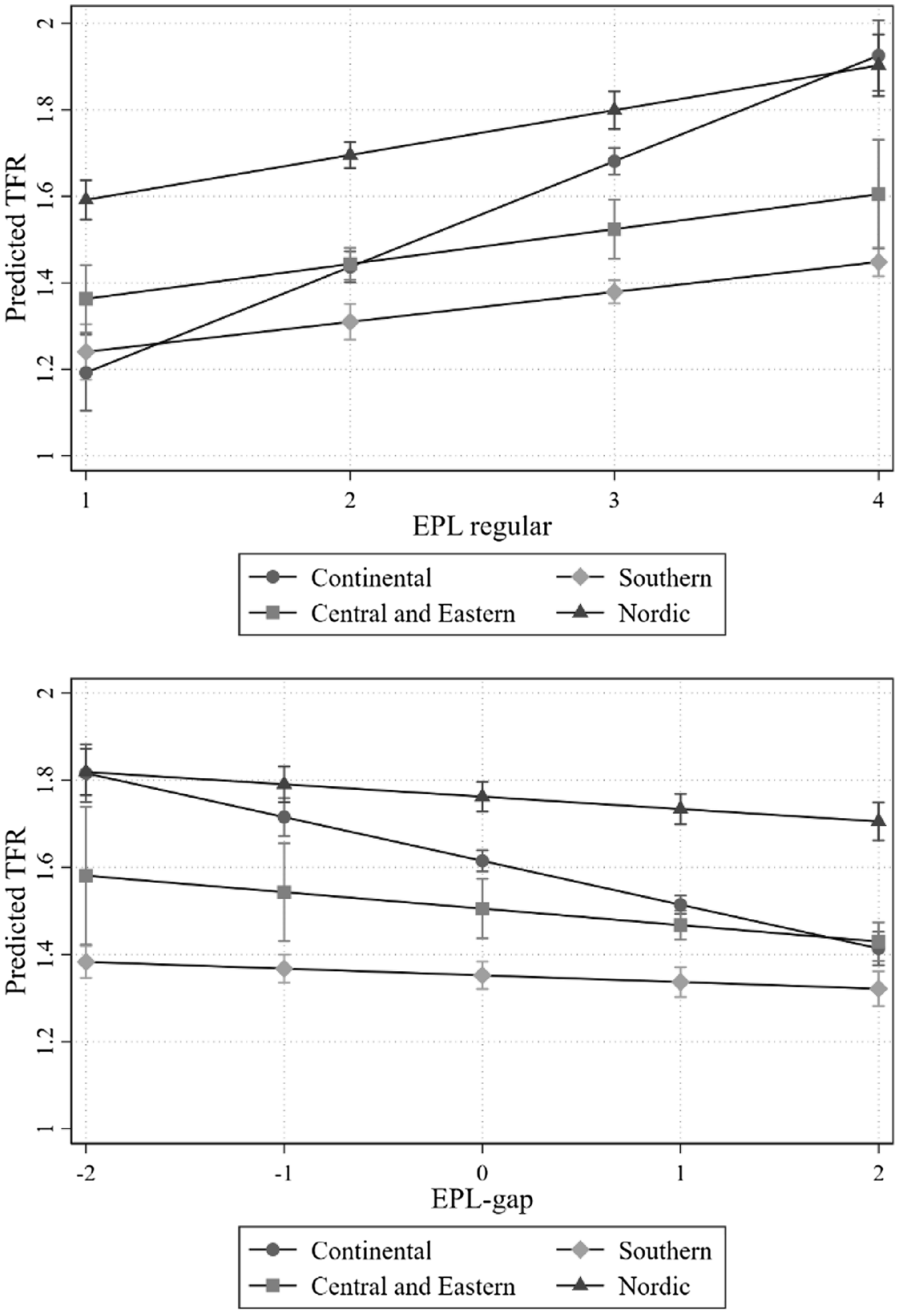
Interaction of EPL-r (top figure) and EPL-gap (bottom figure) with country-areas. *Note*: results based on Eq. (3). Full model results are shown in Table 5 in the appendix. Continental countries include Austria, Belgium, Germany, France, and the Netherlands; Nordic countries include Denmark, Finland, Norway, and Sweden; Southern countries include Italy, Greece, Portugal, and Spain; and Central and Eastern European countries include the Czech Republic, Hungary, Slovakia, and Poland

Finally, the predicted TFRs for each group of countries at different levels of EPL-r and EPL-gap, based on Eq. (3), are displayed graphically in Fig. 2 (full model results can be found in Table 5 in the appendix). Besides Anglo-Saxon countries (not shown),^11^ for all other country groups, we found an increase in TFR as EPL-r increases. Likewise, as labour market dualism increases, the predicted TFR decreases for all country groups (although the reduction in TFR is not statistically significant for Central and Eastern European countries). Overall, results suggest that the pattern described by our hypotheses 1 and 2 holds across different institutional and cultural contexts.

### Robustness Checks

Our main results hold after a series of robustness checks. First, we tested the model in Eq. (1) substituting the EPL-gap with the EPL for temporary contracts. The coefficient of EPL-t is identical to the one of the EPL-gap, although with an opposite sign. In fact, if changes in EPL-r are controlled for, changes in the EPL-gap are given by changes in EPL-t. However, when using EPL-t instead of the EPL-gap the coefficient of EPL-r is slightly reduced in magnitude. Indeed, simultaneously controlling for EPL-r and EPL-gap is similar but it is not the same as controlling for the two EPL indicators separately. Moreover, we tested, instead of the EPL-gap built as the simple difference EPLr − EPLt, the relative gap in EPL = (EPLr − EPLt)/EPLr. Results proved stable also in this different specification. Results from these two models are shown in Table 6 in the appendix.

Then, in order to test to what extent the relationship between EPL and fertility is driven by few countries where variations in EPL are particularly marked, we run our models removing countries such as Portugal and Greece. Our results (available upon request) were robust also to this test.

Finally, some of our control variables may be influenced by the EPL (i.e. the unemployment rate and women’s employment rate). Thus, measuring them simultaneously may conceal some of the effects of EPL. Moreover, responses of fertility rates to changing institutional and economic factors might be delayed. Therefore, we ran two additional models to account for the ordering of effects and possible delayed fertility responses, which can be found in the appendix (Table 7). The estimated effect of EPL-gap is stable across all models.

**Table 4:** Descriptive statistics

Variable		Mean	Std. dev.	Min	Max	Observations
TFR	Overall	1.57	0.24	1.13	2.14	*N* = 564
	Between		0.22	1.29	1.93	*n* = 19
	Within		0.12	1.18	2.13	*T* = 29.68
*Age-specific fertility*						
15–24	Overall	0.03	0.01	0.01	0.10	*N* = 545
	Between		0.01	0.02	0.05	*n* = 19
	Within		0.01	0.01	0.09	*T* = 28.68
25–29	Overall	0.10	0.02	0.05	0.16	*N* = 545
	Between		0.02	0.07	0.13	*n* = 19
	Within		0.01	0.06	0.14	*T* = 28.68
30–34	Overall	0.10	0.02	0.04	0.14	*N* = 545
	Between		0.02	0.06	0.13	*n* = 19
	Within		0.01	0.06	0.13	*T* = 28.68
35–39	Overall	0.04	0.02	0.01	0.10	*N* = 545
	Between		0.01	0.03	0.08	*n* = 19
	Within		0.01	0.02	0.07	*T* = 28.68
40+	Overall	0.01	0.00	0.00	0.01	*N* = 545
	Between		0.00	0.00	0.01	*n* = 19
	Within		0.00	0.00	0.01	*T* = 28.68
*Education-specific fertility*						
Low	Overall	0.04	0.01	0.01	0.07	*N* = 154
	Between		0.01	0.02	0.06	*n* = 13
	Within		0.01	0.01	0.05	*T* = 11.85
Mid	Overall	0.06	0.01	0.03	0.09	*N* = 154
	Between		0.01	0.05	0.07	*n* = 13
	Within		0.01	0.04	0.09	*T* = 11.85
High	Overall	0.08	0.02	0.03	0.12	*N* = 154
	Between		0.02	0.04	0.11	*n* = 13
	Within		0.01	0.06	0.10	*T* = 11.85
*EPL*						
EPL-r	Overall	2.46	0.76	1.10	4.83	*N* = 564
	Between		0.74	1.23	4.17	*n* = 19
	Within		0.23	1.43	3.55	*T* = 29.68
EPL-gap	Overall	0.62	1.15	− 2.86	3.14	*N* = 564
	Between		1.01	− 1.07	2.53	*n* = 19
	Within		0.60	− 1.84	1.92	*T* = 29.68
*Control variables*						
Weeks of paid maternity leave	Overall	64.03	53.19	12.9	214	*N* = 564
	Between		52.86	16	165.11	*n* = 19
	Within		15.29	− 12.92	138.78	*T* = 29.68
Public spending on the family	Overall	2.23	0.93	0.30	4.39	*N* = 517
	Between		0.89	0.94	3.60	*n* = 19
	Within		0.33	1.19	3.43	*T* = 27.21
% Unemployment	Overall	8.70	4.49	2.01	27.47	*N* = 547
	Between		3.38	4.09	17.14	*n* = 19
	Within		3.06	− 0.21	22.53	*T* = 28.79
% Women employment	Overall	50.72	7.08	32.46	63.24	*N* = 564
	Between		6.35	37.30	60.31	*n* = 19
	Within		3.37	38.33	59.31	*T* = 29.68

In the “overall” row, “min” and “max” indicate the minimum and maximum value of the variable in the sample among all countries and years. In the “between” row, “min” and “max” indicate the minimum and maximum country mean of all time points. In the “within” row, “min” and “max” refer to the minimum and maximum difference between the country mean of the variable and the variable in one single year, i.e. the minimum and maximum deviation from the country mean, plus the overall mean (thus, to get the original differences, it is needed to subtract the overall mean from the min and max values)

Variables and sources:

TFR: OECD fertility database https://data.oecd.org/pop/fertility-rates.htm;

Age-specific fertility: Eurostat https://ec.europa.eu/eurostat/databrowser/view/DEMO_FRATE/default/table?lang=en&category=demo.demo_fer

Education-specific fertility: Eurostat https://ec.europa.eu/eurostat/databrowser/view/DEMO_FAEDUC/default/table?lang=en&category=demo.demo_fer

EPL index: OECD https://www.oecd.org/employment/emp/oecdindicatorsofemploymentprotection.htm;

Unemployment rate and Women’s employment rate (% of employed women over the total number of women of working age 15–65): OECD https://data.oecd.org/

Weeks of paid maternity leave: OECD Family database https://stats.oecd.org/index.aspx?queryid=54760

Public spending on the family (as % of GDP): OECD Social expenditure database https://stats.oecd.org/Index.aspx?DataSetCode=SOCX_DET#

## Conclusions

Fertility decline is a matter of major concern on the European agenda, as most EU countries have long been marked by low fertility rates, with negative implications for population ageing and welfare systems. After a rebound in fertility rates in the early 2000s, in the last decade, fertility has again dropped in most European countries. Understanding whether and how labour market flexibilization reforms influence fertility trends is, therefore, of remarkable importance.

This paper evaluates the fertility consequences of the several waves of labour market deregulation reforms implemented in Europe starting from the beginning of the 1990s. We found that, overall, a more regulated and protected labour market is beneficial for fertility while the gap between the regulation of regular and temporary contracts, that is, labour market dualism, hinders fertility plans.

Increasing job protection leads to higher fertility for all age groups, and especially for the youngest ones (aged below 30). In the same fashion, labour market dualism is detrimental to the fertility of all age groups, except for the youngest (aged below 24) for which the effect is virtually null. Nevertheless, at very young ages, fertility rates are very low in European countries, and their determinants are likely to be different (Caldas & Pounder, 1990; Kahn & Anderson, 1992). In addition, we found the positive effects of increased protection for regular contracts and the negative effect of increased labour market dualism on fertility to be relatively homogeneous across European contexts, although stronger in Continental countries.

Breaking down fertility by education, we found that stricter labour market protection for regular contracts positively affect the fertility of all educational groups, while the negative effect of labour market dualism on fertility is especially pronounced among lower-educated women. The lower educated are indeed more likely to be entrapped in the secondary labour market (Garibaldi & Taddei, 2013), and a higher insider–outsider labour market divide may increase their feelings of job insecurity (Balz, 2017).

Our paper has several limitations. First, our main explanatory variables, the OECD EPL indexes, heavily rely on subjective assessments. For this reason, despite their extensive use in scientific research and policy evaluation, they have been widely criticized (Bertola et al., 2000; Boeri & Jimeno, 2005; Myant, 2016). Notwithstanding the extensive efforts undertaken by the OECD to make the indicators valid and reliable (OECD, 2004, 2013, 2019), a considerable degree of arbitrariness goes into the determination of individual scores, which can make cross-country comparability problematic. Another potential issue is that EPL indexes rely on formal legislation, which may not be enforced or may be enforced unevenly. Finally, EPL is limited to formal employment, making it problematic as a measure of de facto labour market regulation in countries with a large informal sector (Myant, 2016). Nevertheless, these issues are a matter of concern mainly when considering cross-country differences in the *levels* of regulation, while in our within-country approach, we partially account for them. Indeed, with our analytic strategy, we removed all between-country heterogeneity and focused on *changes*, thus unambiguously identifying the effects of policy reforms. Second, the analysis does not cover all European Union countries but is limited to those for which data on EPL and fertility are available. In particular, we acknowledge that our results on education-specific fertility are based on a limited number of cases (only 13 countries observed for 11 years). Thus, more research is needed to evaluate differences in the effects of labour market deregulation reforms on the fertility of different educational groups. Third, although we accounted for interactions between EPL and country-areas, further research is needed to delve into the analysis of country-specific differences concerning the relationship between EPL and fertility. Finally, despite the literature reports that changes in EPL affect differently women’s and men’s employment chances, due to our macro-level approach we are not able to disentangle the effects of these gendered processes on aggregate fertility.

Despite these limitations, by analysing the effects of changes in labour market (de)regulation on fertility over the last 30 years, our study reconciles the ambiguous conclusions of existing studies on the impact of employment protection legislation on fertility in Europe. Indeed, it shows that increasing the gap between the regulations of regular and temporary employment through marginal EPL reforms has a negative impact on fertility, especially among low-educated women. On the contrary, increasing employment protection per se has positive effects on fertility, irrespective of age and education. Even if effect sizes are often of small-to-moderate intensity, our results highlight the detrimental consequences of increasing labour market dualism on the reproductive behaviours of Europeans. Policymakers should therefore consider that increasing labour market deregulation and dualism may negatively impact fertility rates.

## Appendix

See Table 4.

### Additional Analyses and Robustness Checks

See Tables

**Table 5:** Interaction of EPL-r and EPL-gap with country-areas

Variables	Interaction of EPL-r and EPL-gap with country-areas
*Area * EPL-r*	
Anglo-Saxon	− 1.130***
	(0.172)
Continental	0.244***
	(0.040)
Southern	0.069***
	(0.019)
Central and Eastern	0.081*
	(0.045)
Nordic	0.104***
	(0.024)
*Area * EPL-gap*	
Anglo-Saxon	0.301***
	(0.088)
Continental	− 0.101***
	(0.017)
Southern	− 0.015*
	(0.008)
Central and Eastern	− 0.038
	(0.034)
Nordic	− 0.028**
	(0.013)
Weeks of paid maternity leave	− 0.001***
	(0.000)
Public spending on the family	0.077***
	(0.014)
% Unemployment	− 0.002
	(0.002)
% Women employment	0.002
	(0.001)
Country-area fixed effects	Yes
Year fixed effects	Yes
Observations	501
*R*-squared	0.797

Standard errors in parentheses. ****p* < 0.01, ***p* < 0.05, **p* < 0.1. Continental countries include Austria, Belgium, Germany, France, and the Netherlands; Nordic countries include Denmark, Finland, Norway, and Sweden; Southern countries include Italy, Greece, Portugal, and Spain; and Central and Eastern European countries include the Czech Republic, Hungary, Slovakia, and Poland. All dependent variables are 1-year lagged relatively to the TFR. The reported coefficients relative to Area * EPL-r and Area * EPL-gap correspond to the effects of EPL in the different areas (main effect + interaction terms)

5,

**Table 6:** Models with EPL-t and relative EPL-gap

Variables	Main	EPL-t	Relative Gap
EPL-r	0.137***	0.100***	0.128***
	(0.035)	(0.033)	(0.034)
EPL-gap	− 0.037***		
	(0.013)		
EPL-t		0.037***	
		(0.013)	
Relative EPL-gap			− 0.089***
			(0.029)
Country-specific time trends	Yes	Yes	Yes
Observations	545	545	545
*R*-squared	0.625	0.625	0.626
Number of countries	19	19	19

Standard errors in parentheses ****p* < 0.01, ***p* < 0.05, **p* < 0.1. EPL variables are 1-year lagged relatively to the TFR

6 and

**Table 7:** Models with different lags on the independent and control variables

Variables	(1)	(2)
	TFR year *T*	TFR year *T*
	EPL year *T* (Jan 1st)	EPL year *T* − 1 (Jan 1st)
	Controls year *T* − 1	Controls year *T* − 2
EPL-r	0.018	0.011
	(0.031)	(0.032)
EPL-gap	− 0.039***	− 0.041***
	(0.012)	(0.012)
Weeks of paid maternity leave	0.001**	0.001*
	(0.000)	(0.000)
Public spending on the family	0.165***	0.105***
	(0.015)	(0.015)
% Unemployment	− 0.015***	− 0.012***
	(0.001)	(0.001)
% Women employment	0.012***	0.011***
	(0.003)	(0.004)
Country-specific time trends	Yes	Yes
Observations	501	499
*R*-squared	0.723	0.723
Number of countries	19	19

Standard errors in parentheses ****p* < 0.01, ***p* < 0.05, **p* < 0.10. In model 1, TFR is measured at year *T*, EPL-r and EPL-gap are measured at January 1st of that year, and the control variables are measured at year *T* − 1. In model 2, TFR is measured at time *T*, EPL-r and EPL-gap are measured at January 1st of the previous year, and the control variables are lagged by 2 years

7.

## Footnotes

1 We use the term “regular contracts” for permanent/open-ended contractual forms as defined by the OECD: https://www.oecd.org/els/emp/oecdindicatorsofemploymentprotection.htm.

2 For other EU countries, data on EPL were not available (Bulgaria, Cyprus, Romania, and Malta) or were available only for a few recent years (since 2018 for Estonia, Luxemburg, and Slovenia; since 2012 for Latvia; since 2014 for Lithuania; only in 2015 for Croatia).

3 Data for Slovakia and the Czech Republic are available beginning in 1993.

4 For Austria, the data are only available through 2012, and for Belgium, through 2014.

5 The overall correlation between EPL-r and EPL-t is 0.32, while the within-country correlation is 0.23.

6 Public spending on the family includes child-related cash transfers to families with children, public income support payments during periods of parental leave, income support for single-parent families, public spending on services for families with children (e.g. childcare and public spending on assistance for young people) and financial support for families provided through the tax system.

7 In our data, Anglo-Saxon countries include the UK and Ireland; Continental countries include Austria, Belgium, Germany, France, and the Netherlands; Nordic countries include Denmark, Finland, Norway, and Sweden; Southern countries include Italy, Greece, Portugal, and Spain; and Central and Eastern European countries include the Czech Republic, Hungary, Slovakia, and Poland.

8 This model specification implies that both differences *between* and *within* countries in each European area contribute to the estimated effects of EPL-r and EPL-gap. While this specification exposes our findings to possible country-specific unobserved heterogeneity, if the results align with those of the FE models, they allow for higher generalizability of our main findings.

9 The z statistics confirm that the effect of EPL-r for the 25–29 group is significantly stronger than for the 35–39 and 40 + age groups (at the 5% level). Differences are also statistically significant between the 15–24 and 40 + age groups.

10 The z-test shows that differences between the 30–34 and the other age groups are statistically significant at the 5% level.

11 The Anglo-Saxon group is only constituted by the UK and Ireland, which undertook limited changes in EPL during our observational window (see Fig. 1).
